# A Comparison of the Modified Alvarado Score and the Raja Isteri Pengiran Anak Saleha Appendicitis (Ripasa) Score in a Southeast Asian Population With Histopathology as the Gold Standard

**DOI:** 10.7759/cureus.46715

**Published:** 2023-10-09

**Authors:** Hummaz Mehbub, Aftab A Baig, Rizwan Khalid, Muhammad Shahid Mehmood, Obaid Ur Rehman, Usman Ghani, Ashfaq Ahmad

**Affiliations:** 1 General Surgery, Akhtar Saeed Medical and Dental College, Lahore, PAK; 2 General Surgery, Allama Iqbal Medical College, Lahore, PAK

**Keywords:** mas vs. ripasa, alvarado vs. ripasa, modified alvarado vs. ripasa, modified alvarado vs. ripasa comparison, southeast asian, acute appendicitis diagnosis, appendicitis, acute appendicitis, ripasa score, modified alvarado score

## Abstract

Background

The diagnosis of acute appendicitis has remained difficult despite it being one of the most common surgical emergencies in the world. One of the most frequently used scoring systems is the Modified Alvarado Score (MAS). However, the MAS has been known to be less efficient in Asian populations. To overcome this issue, the Raja Isteri Pengiran Anak Saleha Appendicitis (RIPASA) score has been specifically developed to improve the diagnosis of acute appendicitis in Asian populations. This study aimed to evaluate the accuracy of the RIPASA score compared to the MAS for the diagnosis of acute appendicitis in a Southeast Asian population keeping histopathology as the gold standard.

Methodology

The study group comprised 150 patients. Data were collected from each patient using a simple proforma to ascertain both the MAS and the RIPASA score for each patient at the time of presentation. The patients then underwent open appendectomy and histopathology was used as the gold standard to determine the presence or absence of acute appendicitis in the excised specimens.

Results

The RIPASA score had a sensitivity and specificity of 89.83% and 59.38%, respectively, compared to 64.41% and 53.12%, respectively, for the MAS. Diagnostic accuracy was similarly higher for the RIPASA score at 83.33% versus 62.00% for the MAS.

Conclusions

The RIPASA score is superior to the MAS for the diagnosis of acute appendicitis. Using the RIPASA score instead of the MAS in Southeast Asian populations can lead to a more accurate and timely clinical diagnosis of patients with suspected acute appendicitis and help improve patient outcomes.

## Introduction

Reginald H. Fitz was the first to use the word Appendicitis in a paper published in 1886 [1], in which he identified the appendix as the most prevalent cause of right lower quadrant inflammatory illness and described the clinical symptoms of the condition as well as the need for early surgical management.

Since then, the diagnosis of acute appendicitis has long been a source of consternation for surgeons across the world. While the approximate incidence of acute appendicitis is around 100 per 100,000 person-years and has been decreasing for unknown reasons since the 1970s [2], appendicitis remains one of the most common abdominal emergencies requiring surgical intervention, with a lifetime prevalence rate of approximately 14% [3]. Determining whether a patient requires emergency surgery, admission and observation, or further evaluation and workup can be a challenge, even with the availability of various diagnostic tools and scoring systems, and a significant number of patients still experience a delayed or missed diagnosis.

As the disease is most frequently a clinical diagnosis and confirmation can only be achieved on histopathology of the excised specimen, it is important to identify all such patients who present with the disease as early in their clinical course as missed diagnoses lead to increased adverse outcomes. Simultaneously, it is necessary to reduce the rates of negative laparoscopies/laparotomies, as these lead to increased patient morbidity, failure to treat the underlying cause, and an increase in the burden and cost of healthcare in hospitals.

Multiple scoring systems have been developed over the years to help support clinicians in making a more accurate diagnosis, such as the Alvarado Score and Modified Alvarado Score (MAS), the Paediatric Appendicitis Score, Tzanakis Score, Lintula Score, and Fenyo-Lindberg Score, to name a few. Of these, the MAS is the one most frequently used in most clinical settings to aid diagnosis.

The Alvarado Score was developed in 1986 for detecting acute appendicitis in pregnant women and was modified for general use in the form of the MAS. However, as the MAS was refined in Western countries, it has been found to be lacking in the accurate diagnosis of acute appendicitis in Asian populations. To overcome this, a new score was developed by researchers in Brunei Darussalam, named the Raja Isteri Pengiran Anak Saleha Appendicitis (RIPASA) score. It offers better diagnostic accuracy than the MAS in the diagnosis of acute appendicitis.

This study aimed to determine the diagnostic accuracy of the RIPASA score against the MAS in a local Southeast Asian (Pakistani) population with histopathology as the gold standard.

## Materials and methods

This was a cross-sectional study involving 150 patients conducted between January 2021 and June 2022 at Akhtar Saeed Trust Hospital, Lahore, Pakistan. Ethical Committee approval was obtained before the start of our study (IRB approval number: 1216-20-ASTH-GSGR). We included patients of both genders with ages ranging between 10 and 60 years with complaints of pain in the right iliac fossa for less than seven days who had suspected acute appendicitis and who consented to open appendectomy. Patients with comorbidities (pregnancy, diabetes mellitus, ovarian/abdominopelvic mass, renal tract stones, carcinomas), perforated appendicitis, presence of appendicular mass formation, or signs and symptoms of local and/or generalized peritonitis were excluded from our study.

The MAS and RIPASA score were then calculated for all patients who fit the inclusion criteria. If the MAS was ≥7 and/or the RIPASA score was ≥7.5, the patient was deemed as having suspected acute appendicitis and advised admission and open surgery. Conversely, if the MAS was <7 and/or the RIPASA score was <7.5, the patient was admitted for observation and conservative management. If a patient did not improve clinically in 48-72 hours or deteriorated, then he/she was advised for open surgery as well. Informed consent was taken from each patient and/or their legal guardian(s) before surgery.

The excised specimen was collected under aseptic conditions in a specimen container containing a preserving agent, was properly labeled, and sent immediately to the Pathology Department for histopathological evaluation and reporting. The presence of neutrophilic infiltration of the muscularis propria was taken as a positive finding for confirmatory diagnosis. The data gathered using the proforma and the final histopathology report for each patient were then recorded and correlated to determine the sensitivity, specificity, positive predictive value (PPV), and negative predictive value (NPV), as well as the diagnostic accuracy for each scoring system.

Statistical analysis was performed using SPSS version 26 (IBM Corp., Armonk, NY, USA) and MedCalc for Windows, version 20.115 (MedCalc Software, Ostend, Belgium). Two-by-two tables were generated to measure the sensitivity, specificity, PPV, NPV, and diagnostic accuracy of each of the scoring systems.

## Results

A total of 150 patients were included in our study (N = 150). Patient demographics are presented in Table 1 below. In total, 83 (55%) of the 150 patients were female and 67 (45%) were male. The mean age was 28.0 ± 8.7 years. A total of 22 (14.7%) patients were between 11 and 20 years of age, 79 (52.7%) patients were between the ages of 21 and 30 years, 38 (25.3%) were between 31 and 40 years of age, and 11 (7.3%) were between the ages of 41 and 50 years.

**Table 1:** Patient demographics.

N = 150	n	%
Male	67	45%
Female	83	55%
Age 11–20 years	22	14.7%
Age 21–30 years	79	52.7%
Age 31–40 years	38	25.3%
Age 41–50 years	11	7.3%

Table 2 shows an analysis of the presenting signs and symptoms. Overall, 139 (92.7%) patients were below the age of 40 years. In total, 91 (60.7%) patients had migratory pain at the time of presentation. Further, 51 (34%) had anorexia and 71 (47.3%) had nausea/vomiting. In total, 67 (44.7%) patients exhibited rebound tenderness, 93 (62%) had abdominal guarding, and 42 (28%) had a positive Rovsing sign. Of the 150 patients, 79 (52.7%) had a positive urinalysis. In total, 138 (92%) patients had a fever and 140 (93.3%) had leucocytosis. Further, 115 (76.7%) patients had symptoms for fewer than 48 hours.

**Table 2:** Signs and symptoms. RIF = right iliac fossa

N = 150	n	%
Age ≤40 years	139	92.7%
Migratory pain	91	60.7%
Anorexia	51	34%
Nausea/Vomiting	71	47.3%
Rebound tenderness	67	44.7%
Guarding in RIF	93	62%
Positive Rovsing sign	42	28%
Positive urinalysis	79	52.7%
Fever	138	92%
Leucocytosis	140	93.3%
Symptoms for <48 hours	115	76.7%

The MAS was positive (≥7) in 91 out of 150 patients, whereas the RIPASA score was positive (≥7.5) in 119 out of the total 150 patients (Table 3). A total of 118 (78.7%) patients in our study group had a positive diagnosis of acute appendicitis on histopathology.

**Table 3:** Evaluation of scoring systems and histopathology. RIPASA score = Raja Isteri Pengiran Anak Saleha Appendicitis score; MAS = Modified Alvarado Score

N = 150	n	%
Positive RIPASA (≥7.5)	119	79.3%
Positive MAS (≥7)	91	60.7%
Positive histopathology	118	78.7%

Two-by-two tables were constructed for calculating sensitivity, specificity, PPV, NPV, and diagnostic accuracy. Upper and lower limits with 95% confidence intervals (CIs) were also calculated for each statistic.

Of the 91 patients who had a positive MAS, 76 (77.7%) patients were found to have a positive histopathology as well (Table 4). Whereas 42 (66.7%) out of 59 patients who had a negative MAS were identified as having acute appendicitis on histopathology.

**Table 4:** Modified Alvarado Score evaluation.

		Histopathology		Total
		Positive	Negative	
Modified Alvarado Score	Positive	76	15	91
	Negative	42	17	59
Total		118	32	150

This study was able to achieve a sensitivity of 64.41% and a specificity of 53.12% for the MAS. PPV and NPV were 83.52% and 28.81%, respectively. A diagnostic accuracy of 62% was calculated (Table 5).

**Table 5:** Statistical analysis of the Modified Alvarado Score. *: upper and lower limits with 95% CI calculated using MedCalc for Windows, version 20.115 (MedCalc Software, Ostend, Belgium). CI = confidence interval; PPV = positive predictive value; NPV = negative predictive value

Statistic	Estimate	Lower to upper limits (95% CI)*
Sensitivity	64.41%	55.07% to 73.00%
Specificity	53.12%	34.74% to 70.91%
PPV	83.52%	77.39% to 88.24%
NPV	28.81%	21.24% to 37.79%
Diagnostic accuracy	62.00%	53.72% to 69.79%

Of the 119 patients, 106 (89.1%) who had a positive RIPASA score were identified as having acute appendicitis on histopathology (Table 6). Of the 31 patients who had a negative RIPASA score, 12 (38.7%) patients had a positive histopathology for acute appendicitis.

**Table 6:** RIPASA score evaluation. RIPASA score = Raja Isteri Pengiran Anak Saleha Appendicitis score

		Histopathology		Total
		Positive	Negative	
RIPASA score	Positive	106	13	119
	Negative	12	19	31
Total		118	32	150

For the RIPASA score, this study achieved a sensitivity of 89.83% and a specificity of 59.38%. PPV was 89.08% and NPV was 61.29%. Diagnostic accuracy was 83.33% (Table 7).

**Table 7:** Statistical analysis of the RIPASA score. *: upper and lower limits with 95% CI calculated using MedCalc for Windows, version 20.115 (MedCalc Software, Ostend, Belgium). RIPASA score = Raja Isteri Pengiran Anak Saleha Appendicitis score; CI = confidence interval; PPV = positive predictive value; NPV = negative predictive value

Statistic	Estimate	Lower to upper limits (95% CI)*
Sensitivity	89.83%	82.91% to 94.63%
Specificity	59.38%	40.64% to 76.30%
PPV	89.08%	84.23% to 92.57%
NPV	61.29%	46.29% to 74.41%
Diagnostic accuracy	83.33%	76.39% to 88.91%

Negative appendectomy rates were calculated to be 10.91% for the RIPASA score and 16.48% for the MAS.

## Discussion

Acute appendicitis is most often a clinical diagnosis, and confirmation can only be achieved by histopathology of the excised specimen. Therefore, it is imperative to identify all patients with the disease as early in their clinical course as possible, as missed diagnoses can result in adverse patient outcomes. At the same time, it is necessary to reduce the rates of negative laparoscopies/laparotomies, as these lead to increased patient morbidity, failure to treat the underlying cause, and an increase in the burden and cost of healthcare in hospitals.

The original study for the Alvarado Score in 1986 achieved a sensitivity of 81% and a specificity of 74% [4]. An important utility of the Alvarado Score lies in its ability to rule out appendicitis, as opposed to indicating the likelihood of appendicitis. A low Alvarado Score of <4 has been noted to have a greater diagnostic utility to exclude a diagnosis of appendicitis [5]. A review of 42 prospective and retrospective studies of over 8,300 patients showed that a score of >4 was found in 99% of patients with acute appendicitis [6].

The Alvarado Score was adapted for general use in the form of the MAS. A study by Nasiri et al. on the MAS calculated a sensitivity of 65.7% and a specificity of 37.5% using a score of 7 as the cut-off point [7]. Meltzer et al. calculated the sensitivity and specificity of the MAS to be 72% and 54%, respectively [8]. Kasabe et al. arrived at a sensitivity of 89.17% and a specificity of 44.44% for the MAS [9].

The RIPASA score takes its name from the Raja Isteri Pengiran Anak Saleha Hospital in Brunei Darussalam where a team of medical researchers came up with the idea of developing a new scoring system for the clinical diagnosis of acute appendicitis that was specifically tailored for Asians, as the Alvarado Score and the MAS were proven to be poorly adapted to South Asian populations based on ethnic differences [10,11].

The pioneering research by Chong et al. in 2010 was a retrospective study with data gathered from 312 patients who had undergone an emergency appendectomy as their primary surgical procedure between October 2006 and May 2008 [12]. A panel of surgeons at the hospital decided on 14 parameters of assessment, with one additional parameter specific to their local population. These 14 parameters now form the criterion for the RIPASA score. The data derived from the study was used to plot a receiver operating curve and derive an optimal cut-off threshold score of 7.5 with a sensitivity and specificity of 88% and 67%, respectively, and PPV and NPV of 93% and 53%, respectively. Their research showed that the new RIPASA score was better at diagnosing acute appendicitis when applied to a South Asian population compared to the Alvarado Score and the MAS. The predicted negative appendectomy rate for the RIPASA score was 6.9%, which was a significant reduction from the 20%-40% rate reported for the Alvarado Score and the MAS. The authors of the study admitted that while the research was done specifically on their local subset of patients, the results were likely applicable to other Southeast Asian and Middle Eastern populations as well.

A second, prospective study by Chong et al. was conducted a year later on a further 192 patients [13]. This time, they were able to reach a sensitivity, specificity, PPV, NPV, and diagnostic accuracy of 98%, 81.3%, 85.3%, 97.4%, and 91.8%, respectively, for the RIPASA score in comparison to 68.3%, 87.9%, 86.3%, 71.4%, and 86.5%, respectively, for the MAS. They concluded that the additional parameters in the RIPASA score made it more flexible and adaptable to different geographical regions.

Multiple regional studies have been able to back these results. A prospective study of 206 patients from India [14], published in 2014, showed higher sensitivity and specificity of the RIPASA score when compared to the MAS (96.2% versus 58.9% and 90.5% versus 85.7%, respectively). Another study from India showed 64.44% sensitivity and 58.82% specificity for the MAS versus 87.78% and 76.47%, respectively, for the RIPASA score [15]. A 2018 study from Nepal was able to arrive at 88.1% sensitivity and 28.8% specificity for the RIPASA score versus 68.6% sensitivity and 28.6% specificity for the MAS [16].

Studies from Jordan, Kuwait, Iran, and Turkey as well as a multicenter, cross-border study between Saudi Arabia and Egypt corroborated the results of the original study in the Middle Eastern population as well [17-21].

After observing positive results from multiple Eastern studies, Malik et al. were among the first to evaluate the RIPASA score in a western population in Ireland. Their retrospective study of 208 patients was able to achieve an 85.93% sensitivity, a 69.86% specificity, and a diagnostic accuracy of 80.01% [22].

A meta-analysis of 12 studies comparing the RIPASA score with the Alvarado Score was published by Greek authors in 2018. It comprised 2,161 patients in total. Sensitivities of the RIPASA and Alvarado Scores were 94% and 69% and specificities were 55% and 77%, respectively. The calculated diagnostic accuracy was 94.3% for the RIPASA score and 79.4% for the Alvarado Score [23].

A few studies have also been conducted in Pakistan to evaluate the effectiveness of the RIPASA score in diagnosing acute appendicitis. Butt et al. were the first to evaluate the RIPASA scoring system in a local population. In a study conducted on 267 patients, they were able to achieve sensitivity, specificity, and diagnostic accuracy of 96.7%, 93%, and 95.1%, respectively [24].

Further studies from Abbottabad, Karachi, Peshawar, and Rawalpindi have been consistent with similar results with high sensitivity, specificity, and diagnostic accuracy, validating the diagnostic prowess of the RIPASA score when compared to other scoring systems [25-30].

Our study was able to replicate the results of these local and international studies and was able to achieve sensitivity and specificity of 89.8% and 59.38% respectively, for the RIPASA score. PPV and NPV were 89.08% and 61.29%, respectively, and diagnostic accuracy was calculated to be 83.33%. For the MAS, our results showed sensitivity, specificity, PPV, NPV, and diagnostic accuracy of 64.41%, 53.12%, 83.52%, 28.81%, and 62%, respectively. The negative appendectomy rate in our study was 10.91% for the RIPASA score versus 16.48% for the MAS.

The sensitivity, specificity, PPV, NPV, and diagnostic accuracy of the RIPASA score were better than those of the MAS. Similarly, the calculated negative appendectomy rate was also lower for the RIPASA score when compared to the MAS. Interestingly, scores of ≥7.5 can be achieved on the RIPASA score without the use of any lab investigations. This fact is of importance as being able to make a rapid diagnosis in the emergency room based on history and clinical examination alone will reduce unnecessary admissions, patient morbidity, and negative appendectomy rates while allowing the attending clinician to make a fairly accurate and early diagnosis of acute appendicitis.

Our study was not without its limitations. A sample size of only 150 patients may not be large enough to generalize the findings to a broader population, although other local and regional studies have also been conducted on a similar number of patients. In addition, the exclusion of patients with comorbidities and other conditions may limit the applicability of the findings to a more diverse patient population. Despite these limitations, the study provides valuable insights into the utility of the MAS and RIPASA score in the diagnosis of acute appendicitis.

## Conclusions

Based on our study findings, the RIPASA score with a cut-off value of ≥7.5 is a superior diagnostic tool compared to the MAS for the diagnosis of acute appendicitis (p ≤ 0.01). It offers better sensitivity, specificity, and diagnostic accuracy and can be calculated relatively easily using patient history, clinical examination, and commonly used lab investigations. These findings suggest that the RIPASA score may be a more reliable tool for diagnosing acute appendicitis in patients with acute right iliac fossa pain. Future research should consider larger sample sizes, longer study durations, and more diverse patient populations to further validate the diagnostic accuracy of these scoring systems and their potential role in guiding clinical decision-making.

## Appendices

**Table 8:** Modified Alvarado Score. RIF = right iliac fossa

Variable	Score
Migration of pain to RIF	☐ +1
Anorexia	☐ +1
Nausea and vomiting	☐ +1
Tenderness in RIF	☐ +2
Rebound tenderness	☐ +1
Elevated temperature >37.3°C or 99.1°F	☐ +1
Leukocytosis	☐ +2
Total	

**Table 9:** Raja Isteri Pengiran Anak Saleha Appendicitis (RIPASA) score. RIF = right iliac fossa; WBC = white blood cell

Variables	Scoring	
Sex	☐ Male +1	☐ Female +0.5
Age	☐≤40 +1	☐ >40 +0.5
RIF pain	☐ Yes +0.5	☐ No 0
Migration of pain to RIF	☐ Yes +0.5	☐ No 0
Anorexia	☐ Yes +1	☐ No 0
Nausea and vomiting	☐ Yes +1	☐ No 0
Duration of symptoms	☐ ≤48 hours +1	☐ >48 hours +0.5
RIF tenderness	☐ Yes +1	☐ No 0
Guarding	☐ Yes +2	☐ No 0
Rebound tenderness	☐ Yes +1	☐ No 0
Rovsing sign	☐ Yes +2	☐ No 0
Temperature between 37°C and 39°C	☐ Yes +2	☐ No 0
Elevated white blood cell count	☐ Yes +1	☐ No 0
Negative urine analysis (absence of blood, WBCs, bacteria)	☐ Yes +1	☐ No 0
Total		
